# The Medical and Surgical Results of the War

**Published:** 1876-08

**Authors:** 


### Editorial.

### The Medical and Surgical Results of the War.

Through the courtesy of the Surgeon General of the Army and of the Chief Medical Purveyor, Dr. J. H Baxter, we have recently been made the recipient of three ponderous volumes of medical interest* The publication of the second volume of the Surgical History of the War has been looked for with eagerness by the profession, and no estimate which they could have formed of its importance and worth can, we think, be disappointed.

While in our accurate scientific attainments in Pathological and Physiological Science, we probably do not equal our brethren in the Old World, it will, we think, be readily admitted that in the practical adaptation of what is of value in medical and surgical science American physicians are surpassed by none. In no department of medicine, we think, is this more clearly shown than in the field of surgery, and to-day, in that branch at least, we yield the palm to none. At the outbreak of the war of the rebellion the medical staff of the army was from necessity, largely recruited from physicians whose entire knowledge of medicine was derived from civil practice. They

*The Medical and Surgical History of the War of the Rebellion, Part II, Vol. II, Surgical History. Prepared under the direction of Joseph K. Barnes. M. D., Surgeon General U. S. Army. By George A. Otis, M. D., Asst. Surg. U. S. A. Washington Government Printing Office, 1876.

Statistics, Medical and Anthropological, of the Provost Marshal General's Bureau, derived from Records of the examination for military service in the armies of the United States during the late war of the rebellion of over a million recruits, drafted men, substitutes and enrolled men. Compiled under the direction of the Secretary of War. By J. H. Baxter, A. M., M, D. In two volumes, Washington Government Printing Office, 1875.

were entirely ignorant, many of them, of the demands of army practice ; hospital management and discipline, the keeping of accurate records and making reports, transportation of the sick and wounded, the proper treatment of gunshot injuries, army hygiene and the thousand and one important duties devolving upon an army surgeon. were to them at best only matters of theoretical knowledge. Out of this raw material was organized a medical corps whose achievements are the admiration of the world, and which to-day is the model of all civilized governments. The results of their labor and observations have been given to the world in the famous circulars which have from time to time been issued by the Surgeon-General's Office, and in the two preceeding volumes of the Medical and Surgical History of the War. The first volume of the surgical portion was devoted to injuries of the head, face neck, spine and chest, of which details were given of over forty-nine thousand cases.

In the present volume, following the same plan inaugurated in volume first, the account of casualities is arranged in relation to their regional classification. In this volume eight thousand five hundred and thirty eight cases of wounds of the abdomen are tabulated, and details of six hundred and ten recorded ; three thousand one hundred cases of wounds of the pelvis are recorded and six hundred and ten detailed ; twelve thousand six hundred and eighty-one cases of flesh wounds of the back are enumerated, and abstracts of two examples are given. Eighty-eight thousand seven hundred and forty-one cases of wounds of the upper extremities are considered, forty-five thousand and eighty-six injuries of soft parts, and thirty-three thousand six hundred and fifty-five cases of shot fractures. Of these detailed abstracts are given of eight hundred and seventeen cases ; the principal facts concerning thirty-seven hundred and twelve excisions, and eighty-two hundred and forty-five amputations are concisely recorded in tabular form.

To attempt any critical analysis of this great work would be a task requiring months of careful study and comparison. It has no rival in the world, in surgical literature ; its compilation marks an era in scientific progress, in the medical record of our nation, in the professional advancement of the world. We can, as members of the medical profession, as brethren of those whose notes of cases and whose arduous duties are here recorded, accord to it no praise which will be too fulsome. To American surgery it is a monument more enduring than granite, to American surgeons a stimulus to still greater perfection in their work.

The two volumes of statistics which have been prepared under the direction of Dr. J. H. Baxter, are of great value, as presenting the results of careful and systematic examinations of a large number of men representing every class of society. In order to make the comparison easy of comprehension, the editor has made use of the plan of expressing the given ratios by the relation that one number bears to another. A large number of charts and tabular views are also introduced, showing at a glance the influence of occupation or condition of life upon disease. Maps are drawn and colored to represent the prevalence of various diseases in certain localities. To attempt an analysis of the facts established by this work far beyond the time or space we have at disposal.

The statistician, the philanthropist and the student of public health will find here a vast field of investigation, a treasure-house of facts. Dr. Baxter should be most heartily thanked by the profession and the public for his patient labor in preparing these vast details in so comprehensive a manner, and also for his intelligent and systematic supervisiou of the Medical Department of the Provost-Marshall-General's Bureau during the war, which made these data attainable, and the present publication a possibility.

These two works, the Medical and Surgical History of the War, and Baxter's Statistics, are a mounment to the intelligence and indefatigable zeal ot the medical department of the army of the United States during the War. They alone are matters of pride and rejoicing to every member of the profession, and taken in connection with the Army Medical Museum, and the Library of the Surgeon-General's office, both creations of but a few years, we are astounded at the amount of work which has been accomplished.

All honor to Surgeon-General Barnes and his able corps of fellow workers, who have done more than any other body of men, to reflect credit on American medical science.

